# Parsing the heterogeneity of depression: a data-driven subgroup derived from cognitive function

**DOI:** 10.3389/fpsyt.2025.1537331

**Published:** 2025-01-30

**Authors:** Chenyang Xu, Yanbao Tao, Yunhan Lin, Jiahui Zhu, Zhuoran Li, Jiayi Li, Mingqia Wang, Tao Huang, Chuan Shi

**Affiliations:** ^1^ Peking University Sixth Hospital, Peking University Institute of Mental Health, NHC Key Laboratory of Mental Health (Peking University), National Clinical Research Center for Mental Disorders (Peking University Sixth Hospital), Beijing, China; ^2^ The First Affiliated Hospital of Xinxiang Medical College, Xinxiang, Henan, China; ^3^ Beijing Key Laboratory of Mental Disorders, National Clinical Research Center for Mental Disorders and National Center for Mental Disorders, Beijing Anding Hospital, Capital Medical University, Beijing, China; ^4^ Ontario Institute for Studies in Education, University of Toronto, Toronto, ON, Canada; ^5^ Department of Epidemiology and Biostatistics, School of Public Health, Peking University, Beijing, China

**Keywords:** depression, cognitive subtype, cluster analysis, heterogeneity, longitudinal study

## Abstract

**Background:**

Increasing evidences suggests that depression is a heterogeneous clinical syndrome. Cognitive deficits in depression are associated with poor psychosocial functioning and worse response to conventional antidepressants. However, a consistent profile of neurocognitive abnormalities in depression remains unclear.

**Objective:**

We used data-driven parsing of cognitive performance to reveal subgroups present across depressed individuals and then investigate the change pattern of cognitive subgroups across the course in follow-up.

**Method:**

We assessed cognition in 163 patients with depression using The Chinese Brief Cognitive Test(C-BCT) and the scores were compared with those of 196 healthy controls (HCs). 58 patients were reassessed after 8 weeks. We used K-means cluster analysis to identify cognitive subgroups, and compared clinical variables among these subgroups. A linear mixed-effects model, incorporating time and group (with interaction term: time × group) as fixed effects, was used to assess cognitive changes over time. Stepwise logistic regression analysis was conducted to identify risk factors associated with these subgroups.

**Results:**

Two distinct neurocognitive subgroups were identified: (1) a cognitive-impaired subgroup with global impairment across all domains assessed by the C-BCT, and (2) a cognitive-preserved subgroup, exhibited intact cognitive function, with performance well within the healthy range. The cognitive-impaired subgroup presented with more severe baseline symptoms, including depressed mood, guilt, suicidality, and poorer work performance. Significant group × time interactions were observed in the Trail Making Test Part A (TMT-A) and Continuous Performance Test (CPT), but not in Symbol Coding or Digit Span tests. Despite partial improvement in TMT-A and CPT tests, the cognitive-impaired subgroup's scores remained lower than those of the cognitive-preserved subgroup across all tests at the study endpoint. Multiple regression analysis indicated that longer illness duration, lower educational levels, and antipsychotic medication use may be risk factors for cognitive impairment.

**Conclusion:**

This study identifies distinguishable cognitive subgroups in acute depression, thereby confirming the presence of cognitive heterogeneity. The cognitive-impaired subgroup exhibits distinct symptoms and persistent cognitive deficits even after treatment. Screening for cognitive dysfunction may facilitate more targeted interventions.

**Clinical Trial Registration:**

https://www.chictr.org, identifier ChiCTR2400092796.

## 1 Introduction

Major Depressive Disorder (MDD) is a heterogeneous clinical syndrome (1, 2) that is diagnosed when a patient meets at least five of the nine symptoms listed in DSM-IV/DSM-5, accommodating multiple symptom combinations. Neuroimaging has greatly enhanced our understanding of mental disorders (3, 4). Previous studies have reported that data-driven analyses of biomarkers, such as brain connectivity, volume, and cortical thickness, can identify biologically distinct subgroups associated with specific cognitive performances (5) and predict clinical outcomes more accurately than traditional diagnostic categories (5–8). However, concerns regarding the cost and availability of MRI in practice (9), along with the test-retest reliability of measures derived from short-duration scans, may present substantial barriers to their clinical implementation (4).

Studies have shown that cognitive performance can be predicted by the brain’s functional connectivity patterns (10–13).Furthermore, cognitive impairment often predicts greater psychosocial dysfunction, including diminished quality of life, and social, occupational and global functioning (14–16). Thus, as a neurocognitive marker, cognitive performance can reflect an individual’s biological features to some extent and provide clinicians with valuable information about functionality. Cognitive assessments are relatively straightforward to administer, and their results are intuitively interpretable (17), making them a valuable tool for understanding the presentation of mental disorders, as conceptualized by the RDoC framework (18).

However, studies of cognitive impairment in MDD exhibit considerable heterogeneity. Most meta-analyses report cognitive deficits in executive function, memory, and attention in patients with MDD, with effect sizes ranging from small to moderate (19–23). Notably, a high degree of heterogeneity in results from meta-analyses is widely reported (19, 23, 24), accompanied with inconsistent findings. A meta-analysis found that working memory performance in patients does not significantly differ from that in healthy controls (24). Additionally, some previous studies have found that depressed adults do not exhibit impairments in any assessed cognitive functions (25, 26). Conflicting results are expected when significant neural heterogeneity exists within depressed patients but is overlooked in conventional group-based analyses (8, 27).

Several attempts have been made to specify more homogenous subgroups within MDD. Subtypes have been proposed based on specific combinations of symptoms, onset, course, or severity (28). Most traditional subtyping methods rely on pattern recognition and categorization derived from distinctions observed in clinical practice (29), yet cognitive function is often not a primary consideration in these schemes. More crucially, studies examining cognitive function across various subtypes of MDD have produced inconsistent findings. For instance, some studies suggest that patients with psychotic MDD have more severe cognitive impairments than those with non-psychotic MDD (30), while others report similar levels of impairment (31–34). This inconsistency suggests that traditional subtyping methods may have limited clinical utility in reflecting the cognitive profiles of individuals with MDD.

Additionally, to effectively leverage advances in neuroscience for understanding disease mechanisms, we need new approaches for patient stratification that recognize the complexity and continuous nature of psychiatric traits, and that are not constrained by current categorical approaches (35). The NIMH’s RDoC framework (36) promotes a research paradigm that begins with existing knowledge of behavior-brain relationships and connects these insights to clinical phenomena. Neurocognition, as an intermediary phenotype within behavior-brain interactions, holds significant potential for this purpose (37).Thus, exploring the heterogeneity of MDD through data-driven approach based on cognitive function may be a valuable avenue of investigation.

An early study supported the concept of subgroups and found that only a minority (<30%) of patients with MDD demonstrated measurable cognitive impairment and if this substantial minority was removed from the group statistical analyses, the significant effect sizes disappear (38). Several cross-sectional studies have reported three subgroups based on data-driven approaches. Pu et al. conducted hierarchical cluster analysis and identified three distinct neurocognitive subgroups: mild impairment, selective impairment, and global impairment (39). Similar findings were obtained through latent class analysis (40) and two-step clustering analysis (41). K-means cluster analysis can handle larger data sets than hierarchical clustering, and it uses random number seeds to ensure the stability of the initial central value (42). Limited longitudinal studies reported more on two subgroups identified by k-means clustering: a cognitively impaired group and a cognitively preserved group, characterizing different neurobiological profiles and allowing predictions of treatment response. Guo et al. discovered these two subtypes in the acute episode phase among MDD patients, and 80% of the patients remained in their original subgroup after six months of treatment (43). In a secondary analysis of a randomized clinical trial involving 1008 patients with MDD, 27% exhibited pre-treatment global cognitive impairment and significantly decreased brain response to a cognitive task, as well as poorer response to standard pharmacotherapy, thereby defining a cognitive biotype in MDD (44).

Consistent evidence indicates that cognition dysfunction may exist independently of depressive symptoms and persist during remission (45–49), contributing to and sustaining psychosocial impairment (50–52). Therefore, there is an urgent need for more longitudinal studies to validate the clinical value of these subtypes and to identify the cognitive trajectory of MDD from its initial onset. Additionally, although numerous standardized neurocognitive tests are available, employing generic neurocognitive tests could aid in considering cognition as a cross-diagnostic dimension (53, 54). Last but not least, the lack of readily administered objective cognitive tests impedes the routine screening of cognitive function in MDD by physicians in clinical practice (55).

Our primary research objectives are as follows: (a) We used a machine learning method, specifically cluster analysis, to identify cognitive subgroups within the broader MDD diagnosis, assessing whether they are distinguished by baseline patterns of clinical symptoms, reduced occupational function, and poorer response to antidepressant; (b) We also aimed to determine the cognitive trajectory of MDD in subgroups following eight weeks of acute phase treatment; (c) We used a digital assessment function, the Chinese Brief Cognitive Test (C-BCT), to assess cognitive function. The C-BCT is a cognitive test developed for schizophrenia, with the advantage of objectivity, rapid administration, and established norms within a healthy Chinese population. It has been used to assess cognitive function of MDD patients in several studies and one prospective cohort study (56, 57).

## 2 Materials and methods

### 2.1 Participants

We recruited 164 patients with depression from the outpatients at Peking University Sixth Hospital. The inclusion criteria included: (1) diagnosed with MDD and in a major depressive episode at the moment, (2) age 18 to 60 years, and (3) able to read and understand Mandarin. The healthy control group contains 196 community volunteers. The exclusion criteria included: (1) history of central nervous system trauma, neurological disorders, or comorbid psychiatric disorders (except for anxiety disorders) and (2) diagnosis of intellectual disabilities or pervasive developmental disorders (3) recent diagnosis of substance abuse or dependence (within the past three months), (4) physical illnesses affecting vision and hearing. Depressed participants with co-morbid anxiety were included to maximize the generalizability of the sample, provided that anxiety was not the primary focus of current treatment. Patients were diagnosed by trained psychiatrists using the Mini International Neuropsychological Interview (M.I.N.I.) (58), a structured psychiatric interview based on the Diagnostic and Statistical Manual of Mental Disorders, Fourth Edition (DSM-IV) criteria.

All participants provided informed consent, and the study was conducted with approval from the local institutional Human Research Ethics Committee, adhering to the National Health and Medical Research Council guidelines for human research.

### 2.2 Procedures and research tools

#### 2.2.1 Procedures

This is an eight-week observational study where all MDD patients received personalized antidepressant medication (SSRIs or SNRIs) prescribed by psychiatrist in an outpatient clinic. The researcher made no treatment-related recommendations and only recorded information about the medication. Demographic information, including sex, age, and education level, was collected for all participants. Patients underwent clinical and cognitive assessments at baseline and after eight weeks of follow-up. A total of 58 patients completed the follow-up; detailed reasons for loss to follow-up are provided in the **Supplementary Materials** (**Supplementary Figure 1**).

#### 2.2.2 Measurements of cognitive function

The C-BCT was used to measure neurocognitive functioning (59, 60). C-BCT was initially developed for clinical trials targeting cognitive assessments in schizophrenia. Recent studies have demonstrated that similar instruments, such as the Brief Assessment of Cognition in Schizophrenia (BACS) (39, 61), can effectively in assess neurocognitive deficits in individuals with MDD.

The C-BCT comprises four tests that assess various cognitive domains: (1) Trail Making Test, Part A (TMT-A): the speed of information processing; (2) Symbol Coding: attention, the speed of information processing, and the executive function of transformation; (3) Continuous Performance Test (CPT): sustained and focused attention; (4) Digit Span: the ability of auditory verbal working memory. Patients with MDD underwent the C-BCT and raw subtest scores were standardized by creating age- and sex- corrected T-scores (59, 60), with higher scores reflecting better cognitive performance. We used single scores from cognitive item in the Hamilton Rating Scale for Anxiety (HAM-A) (62) to assess the level of subjective cognitive impairment, in contrast to objective cognitive performance.

#### 2.2.3 Measurements of clinical features and occupational function

Current symptoms, age of onset, duration of illness, first episode of depression (FED) or recurrent major depression were collected using the M.I.N.I. interview. Evaluation of depression severity was conducted using the 17-item Hamilton Rating Scale for Depression (HRSD-17) (63), and anxiety levels were assessed using the HAM-A. The types and dose of patients’ medication were recorded in detail at baseline and at the end of the eight-week follow-up. When benzodiazepines were used more than 50% of the time in the previous week, the use was considered present and this variable was dichotomized into yes/no (64). Occupational function was assessed by asking patients if they had a break from work or study due to MDD.

### 2.3 Statistical analysis

We used R (Version 4.4.1) and Rstudio (Version 2024.04.2 + 764) to conduct cluster analyses. K-means cluster is one of the most commonly used unsupervised machine learning methods (65). We use the ‘cluster’ package to calculate the silhouette metric and the ‘factoextra’ package to plot the relationship between k and WSS (Total Within Sum of Squares). The optimal solution was selected by convergence across multiple criteria: (1) scree plot elbow method using WSS, (2) silhouette metric, and (3) clusters differ on a maximum number of inputs, while ensuring an adequate number of patients in each cluster.

Considering the autocorrelations among repetitive measurements of the same patient, we used a linear mixed effects model for continuous data. This analysis was also conducted in R (Version 4.4.1) using the ‘glmmTMB package’. The ‘ggplot2’ package was utilized to visualize the estimated mixed effects models. To measure the time effect, we entered the follow-up time (from baseline to the last follow-up appointment) as the fixed effect in the model. Different individuals may be prescribed different antidepressants; therefore, we converted the antidepressant doses to fluoxetine equivalents (mg) and included the ‘subject id, antidepressant’ as a random effect. To investigate group differences and group*time interactions, follow-up time and group (with interaction term: time*group) were entered as fixed effects.

Demographic, clinical, and functional variables were analyzed among resulting clusters within each patient group using one-way analysis of variance (ANOVA), Kruskal-Wallis test, or chi-square when appropriate, with effect-sizes also reported.

Stepwise logistic regression analysis was conducted to identify risk factors associated with each subgroup. Independent variables included age, sex, HRSD-17, HAM-A, Duration of illness, FMD, years of education, antidepressants (yes or no), benzodiazepines (yes or no), and antipsychotics(yes or no). Age of onset was excluded from the analysis due to the collinearity with age and age of onset. We use the ‘stepwise’ method to select variables for inclusion in the model.

## 3 Results

### 3.1 Demographic information

The 163 patients with MDD were 18 to 60 years old, with a median age of 29 years (IQR=16) and 64% were female. Of the 58 patients who participated in the follow-up study, 75% were female, with a median age of 29 years (IQR=15) (**Supplementary Table 1**). No significant differences were found in age (*p* = 0.537) or gender (*p* = 0.063) between patients with MDD and HCs. The MDD group had fewer years of education compared to HCs [15(6) vs. 15(2), p =0.005]. Patients with MDD exhibited moderate depressive symptoms (**Table 1**).

**Table 1:** Demographics and clinical characteristics of MDD and HC.

	MDD N=163	HC N=196	Significance
Female	104 (64%)	106 (54%)	χ2 = 3.465, p = 0.063
Male	59 (36%)	90 (46%)	
Age, y	29 (16)	29.5 (13)	U = 15370.0, p = 0.537
Education level, y	15 (6)	15 (2)	U = 13299.0, p =0.005
HRSD-17 score	19.66 ± 4.99	1.24 ± 2.38	F=2096.917, p<0.001
HAM-A	19.43 ± 5.84	0.67 ± 1.33	F=1904.925, p<0.001

Data are n (%), mean ± SD, or median (IQR); HRSD-17, the 17-item Hamilton Rating Scale for Depression; HAM-A, the Hamilton Rating Scale for Anxiety.

### 3.2 Deriving a cognitive subtype

**Figure 1:**
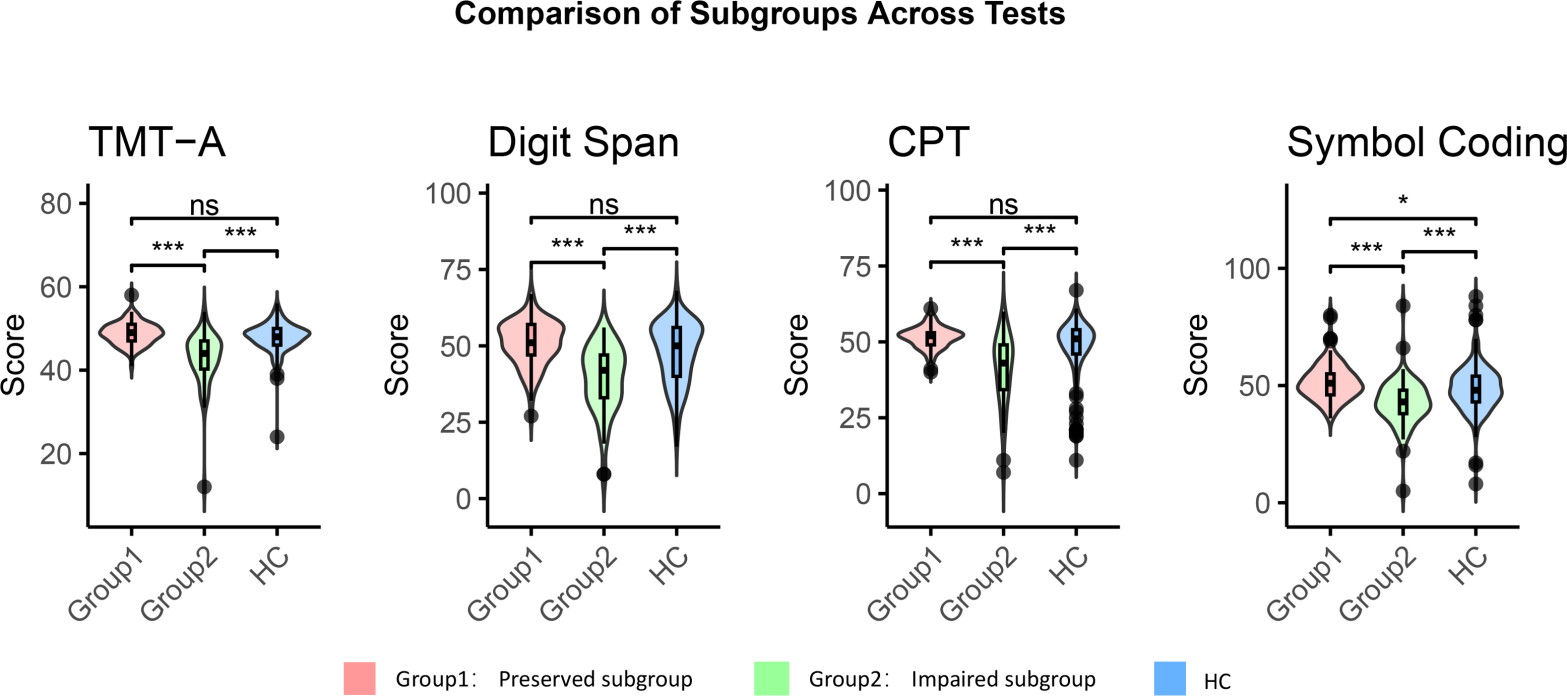
Cognitive function of the cognitive-impaired subgroup, the cognitive-preserved subgroup and HCs. Impaired group, cognitive-impaired subgroup; Preserved group, cognitive-preserved subgroup; HC, healthy control group; CPT, Continuous Performance Test; TMT-A, Trail Making Test, Part A. Comparative analysis was performed using the Mann-Whitney U test; all p values are adjusted for multiple comparisons with Bonferroni correction. * *p*<0.05. *** *p*<0.001. ns *p*>0.05.

The scree plot (**Supplementary Figure 2**) indicates an elbow at two clusters, after which the line flattens, indicating that additional clusters do not contribute to meaningfully separating the data and suggesting k=2 as the most optimal solution. Silhouette scores, which represent the mean silhouette coefficient across all instances of the dataset, range from -1 to1. Higher scores that closer to 1 indicate a model with more coherent clusters. Although the three-cluster solution yielded a silhouette score nearly identical to that of the two-cluster solution (0.32), it was not selected as it did not provide additional explanatory value (**Supplementary Figure 2**). Silhouette scores for k=4 to k=10 clusters were all lower than k=2. Scree plot and the silhouette metric indicated a two-cluster solution was optimal. The two-cluster solution showed significant differences across all cognitive test scores (all *p* <  0.001) (**Figure 1**).

The first cluster, referred to the cognitive-impaired subgroup, was characterized by significant impairments across all cognitive measures, and was present in 40% of individuals. Patients in cognitive-impaired subgroup demonstrated cognitive dysfunction across all tests when compared to HCs (all *p*  < 0 .001, TMT-A: r=0.737; Digit Span: r=0.692; CPT: r=0.675; Symbol Coding: r=0.665) (**Figure 1**). The second cluster, referred to the cognitive-preserved subgroup, exhibited intact cognitive function, with performance well within the healthy range. Notably, this subgroup showed superior cognitive performance to the HCs on the Symbol Coding test (*p* = 0.011,r=0.414) (**Figure 1**). There was no significant difference between the scores of the two groups on the cognitive item in HAM-A, suggesting that both groups have similar levels of subjective cognitive function[2(1) vs. 2(2), *p* = 0.140)], despite differing significantly on all objective cognitive tests.

### 3.3 Baseline symptom profiles and occupation function

The severity of depressive symptoms, as measured by the HRSD-17, was significantly greater in the cognitive-impaired subgroup compared to the preserved subgroup (*p* < 0.001). Regarding individual symptoms, the cognitive-impaired subgroup has several profiles on HRSD-17, including more pronounced depressed mood (*p* =0.016), stronger feelings of guilty (*p* =0.048), higher frequency of suicidality(*p* =0.006), and poorer performance in work and activities (*p* =0.008). Additionally, a higher proportion of patients in the cognitive-impaired subgroup had stopped working due to MDD (**Table 2**).

**Table 2:** Comparison between the two subgroups across demographics, clinical characteristics and occupation function.

	Cognitive-impaired subgroup n=66	Cognitive-preserved subgroup n=97	Significance
Female	42 (64%)	62 (64%)	χ2 = 0.001, p = 0.971
Male	24 (46%)	35 (46%)	–
Age, y	32 (17)	27 (13)	U = 2541.0, p = 0.018
Education level, y	12 (8)	16 (4)	U = 2109.0, p < 0.001
FED, No. (%)	35 (53%)	61 (63%)	χ2 = 1.576, p = 0.209
HAM-A score	21.05 ± 6.24	18.33 ± 5.31	F = 8.912, p = 0.003
Depression Scale Items (HRSD-17)			
Total score	21.56 ± 4.95	18.37 ± 4.62	F=17.687, p < 0.001
Depressed mood	3 (1)	2.5 (1)	U = 2527.5, p =0.016
Feelings of guilty	2 (1)	1 (2)	U = 2635.0, p =0.048
Suicide	2 (2)	1 (3)	U = 2411.0, p =0.006
Work and Activities	2.5 (2)	2 (1)	U = 2456.0, p =0.008
Type of medications			
Antidepressant	31 (46%)	38 (39%)	χ2 = 0.977, p = 0.323
Antipsychotic	13 (20%)	8 (8%)	χ2 = 4.587, p = 0.032
Benzodiazepines	13 (20%)	13 (13%)	χ2 = 1.161, p = 0.281
Occupation function			
Stop working	20 (30%)	13 (13%)	χ2 = 6.948, p = 0.008

Data are n (%), mean ± SD, or median (IQR); FED, First Episode of Depression; HRSD-17, the 17-item Hamilton Rating Scale for Depression; HAM-A, the Hamilton Rating Scale for Anxiety.

### 3.4 Multivariate regression analysis to identify factors associated with cognition clusters

Binomial logistic regression analysis was used to identify the factors associated with cognition clusters. The following factors were included in the multivariate stepwise regression model: HRSD-17 (OR = 1.182, 95% CI [1.088–1.284], *p* < 0.001), duration (OR = 1.200, 95% CI [1.062–1.356], p = 0.003), years of education (OR = 0.878, 95% CI [0.798–0.965], *p* = 0.007), and antipsychotic use (OR = 3.521, 95% CI [1.169–10.606], *p* = 0.025) (**Table 3**), suggesting an association between these factors and the cognitive-impaired subgroup. However, the model’s fit requires further improvement, as the model containing four predictors had an *R^2^* of 0.256, a rescaled *R^2^* of 0.345, a sensitivity of 0.591, and a specificity of 0.845.

**Table 3:** Univariate logistic regression analysis and multivariate stepwise regression analysis.

Clinical variables	Univariate analysis		Multivariate analysis		
	OR (95%CI)	p	OR (95%CI)		p
HRSD-17	1.155 (1.075,1.241)	<0.001	1.182 (1.088,1.284)		<0.001
Duration	1.224 (1.097,1.365)	<0.001	1.200 (1.062,1.356)		0.003
Education level, y	0.841 (0.772,0.916)	<0.001	0.878 (0.798,0.965)		0.007
Antipsychotic	2.678 (1.043,6.880)	<0.001	3.521 (1.169,10.606)		0.025
Sex	0.989 (0.517,1.892)	0.973			
Age	1.042 (1.012,1.073)	0.006			
HAM-A	1.091 (1.031,1.156)	0.003			
FED	1.549 (0.823,2.915)	0.175			
Benzodiazepines	1.707 (0.745,3.912)	0.207			

FED, First Episode of Depression; HRSD-17, the 17-item Hamilton Rating Scale for Depression; HAM-A, the Hamilton Rating Scale for Anxiety; OR, odds ratio; CI, confidence interval.

### 3.5 Cognitive subtype and treatment outcomes at eight weeks

No significant differences were found between the cognitive-impaired and cognitive-preserved subgroups at eight weeks in terms of HRSD-17 score (20.88 ± 4.01 vs. 18.26 ± 5.08, *p* = 0.491), treatment remission (50% vs. 64.7%, *p* = 0.263), and medication use [antidepressant dose (36.75 ± 20.26 vs. 39.19 ± 18.64 mg, *p* = 0.637), antipsychotic use (25.0% vs. 17.6%, *p* = 0.496), and benzodiazepine use (16.6% vs. 38.2%, *p* = 0.076)]. Nearly all patients were able to participate in work at the eight-week follow-up, and there was no significant difference between the two subgroups in the proportion of patients stopping work due to MDD (*p* = 0.230).

**Figure 2:**
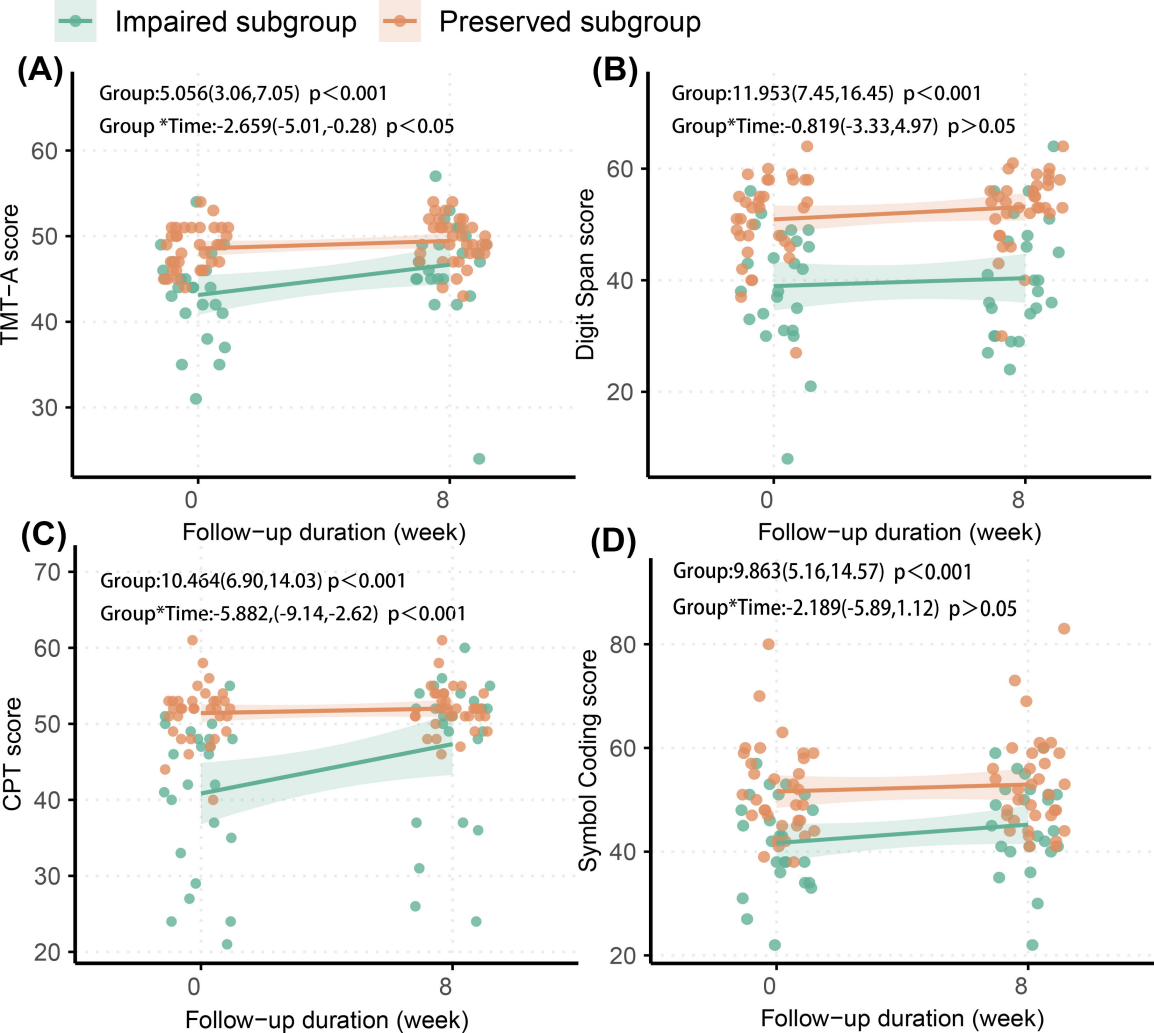
Longitudinal change of cognitive function in two subgroups. **(A)** TMT-A, Trail Making Test, Part A; **(B)** Digit Span; **(C)** CPT, Continuous Performance Test; **(D)** Symbol Coding. The effects (95% confidence interval) of group difference and interaction (group*time) in each figure were estimated in linear mixed effects mode.

Significant group × time interactions were observed in TMT-A and CPT, but not in the Symbol Coding or Digit Span tests (**Figure 2**). These findings suggest that, after eight weeks of acute-phase treatment, the cognitive-impaired subgroup showed improvements in performance on the TMT-A and CPT, but no significant improvement on the Symbol Coding or Digit Span tests. Despite partial improvements in the TMT-A and CPT, the cognitive-impaired subgroup continued to score lower than the cognitive-preserved subgroup across all tests at the study endpoint (all p < 0.05) (**Figure 2**; **Supplementary Table 1**).

## 4 Discussion

In this study, we identified a distinct cognitive-impaired subgroup in MDD using a machine learning clustering algorithm. This subgroup has greater severity in baseline symptoms including depressed mood, feelings of guilty, suicidality and poorer work performance. Despite improvement in depressive symptoms following acute-phase treatment, this subgroup still exhibited significant cognitive impairment.

The present study provides evidence for the existence of cognitive heterogeneity in patients with MDD during the acute episode. And after eight weeks of treatment, the cognitive-impaired subgroup remained preserved, performing worse than the cognitive-preserved group across all cognitive tests, which is consistent with previous longitudinal studies. Recent studies on functional connectivity have identified several fMRI-based biotypes in MDD, which characterize the neurobiological heterogeneity of the disorder and provide insights for personalized treatment. Notably, these fMRI-based biotypes have been shown to correlate with specific cognitive functions. Wen et al. applied a semi-supervised clustering method to regional grey matter (GM) brain volumes and identified two dimensions among patients with late-life depression. Patients in dimension 1 showed relatively preserved brain anatomy without white matter (WM) abnormalities. In contrast, patients in dimension 2 showed widespread brain atrophy and WM integrity deficits, along with cognitive impairment and higher depression severity (66). Similarly, another study identified a subtype in youth with internalizing symptoms, marked by elevated levels of psychopathology, impaired cognition, and multiple deficits apparent on multi-modal imaging (5). It is hypothesized that molecular alterations, along with concomitant changes in neuronal and glial morphometry and integrity, contribute to disruptions within and between brain circuits that are crucial for distinct cognitive domains (67–69). These studies suggest that classifying the cognitive heterogeneity associated with depression may provide a platform for better understanding the neurobiological underpinnings of the disease. Further research is needed to determine the neuroanatomical, neurophysiological, or neuroendocrine abnormalities specific to the cognitive-impaired subgroup.

The cognitive-impaired subgroup seems to have greater severity in negative thinking as assessed by HRSD-17, such as feelings of guilt and suicidality. Previous studies suggest that neuropsychological performance in depression may provide valuable information about risk for suicide. Deficits in interference processing, cognitive control, and memory performance have been found in past suicide attempters (70–72). These deficits are independent of clinical severity measures (70, 71), and residual cognitive deficits following symptomatic remission may contribute to suicide ideation in MDD (73). This highlights the need for greater attention to the safety of patients in the cognitive-impaired subgroup in clinical practice to prevent adverse events. Limited evidence suggests that shared neurobiological mechanisms underlying negative thinking and cognitive functioning may contribute to this relationship. Yang et al. identified a subgroup marked by poorer functioning across multiple cognition domains and increased brain activity in the anterior cingulate cortex and medial prefrontal cortex. This hyper-activation of the default mode could be linked to the Negative Cognition construct (74).

Another key finding of this study is that, even with significant symptom remission, the cognitive performance of the cognitive-impaired subgroup remained poorer than that of the cognitive-preserved subgroup across all tests. Several previous systematic reviews and meta-analyses have indicated that significant residual cognitive impairment persists during the remission phase of depression, including deficits in attention, learning and memory, working memory, and executive function (46, 47, 49). However, the effect sizes of these impairments appear to range from small to moderate, and the heterogeneity between studies should not be overlooked (45, 46). Differences between studies are not surprising when heterogeneity in cognition during remission persists and studies use diagnosis alone as inclusion criterion.

The improvement in CPT and TMT-A tests in the cognitive-impaired subgroup suggests partial recovery in processing speed and sustained visual attention following treatment. A meta-analysis involving 4,639 patients with MDD indicated a modest improvement in sustained visual attention and processing speed after treatment (45). Another meta-analysis comprising 33 studies found that antidepressants have a modest, positive effect on divided attention (75). However, the degree of improvement was insufficient to fully resolve these deficits. There was no improvement in Symbol Coding and Digit Span tests following pharmacological treatment, indicating that medication has limited impact on working memory and executive function related to transformation. Previous studies have suggested that antidepressants do not significant effect on working memory in patients with depression (75). Impaired working memory may contribute to rumination and difficulty breaking habitual thought patterns, thereby hindering effective reappraisal and problem-solving (76). Executive functioning was identified as the strongest independent predictor of functioning in remitted MDD patients (51). Residual cognitive deficits may contribute to ongoing occupational and social dysfunction (67, 77, 78). Furthermore, the persistence of cognitive impairment may interact with pre-existing emotional and social vulnerabilities, elevating the risk of recurrent depressive episodes (79, 80). Given the limited effectiveness of pharmacological treatment in improving cognitive function (81), combining other therapeutic approaches for patients in cognitive-impaired subgroup, such as cognitive rehabilitation training (82, 83) should be considered. Additionally, the use of vortioxetine may be a viable strategy, as it has demonstrated more definitive effects in improving cognitive function (84–87).

The results of multiple regression analysis identified longer duration of illness, lower educational attainment, and the use of antipsychotic medications as risk factors for cognitive impairment. Previous studies have similarly reported the negative effects of illness duration and education level on cognition (48, 88). While the effect of antipsychotic medication use is evident, we did not measure cognitive function before the initiation of the antipsychotics, leaving it unclear whether the medications use itself is merely indicative of underlying cognitive impairment risk or whether the medications contribute directly to cognitive dysfunction (e.g., through extrapyramidal side effects that affect cognition) (89). In this study, no significant impact of recurrence on cognitive function was found, despite previous research indicating that the number of depressive episodes is an important factor influencing cognitive function in depression patients (48, 49). This discrepancy may be due to our study simply categorization of patients as either first-episode or recurrent without accounting for the actual number of episodes. Additionally, the fit of the multiple regression models requires further improvement. Relying solely on these clinical risk factors may result in a relatively high false-negative rate, potentially leading to missed diagnoses of cognitive impairment. Therefore, it remains essential to conduct screening for cognitive function in patients with depression.

Our findings further support that subjective cognition may not accurately reflect objective cognitive function (73, 90). As a digital measurement tool, C-BCT offers a quick, easy-to-administer, and remotely accessible method for cognitive assessment in clinical practice. It covers various domains, including attention, working memory, information processing speed, and executive function. Its applicability across a range of diseases (56, 57, 60) also positions it a potential tool for cross-diagnostic cognitive assessments (53). As the C-BCT was originally designed to assess cognition in patients with schizophrenia, the use of a more comprehensive neurocognitive battery in patients with affective disorders (91, 92), including measures of affective processing, will be important in establishing and refining these cognitive profiles in future studies.

There are several limitations to our findings. First, the high dropout rate during the follow-up period may have resulted in an overrepresentation of patients in remission (the cognitive-impaired subgroup: 50%, the cognitive-preserved subgroup: 64.7%), which could affect our assessment of cognitive patterns during the remission phase. Second, although we considered the impact of medications, the relatively small sample size during follow-up prevented further analysis of medication dosage and type. Considering the abundant evidence of the deteriorating effect of anxiolytics on cognitive function (93–95), the effect of medication cannot be ignored. Third, our cognitive assessments did not include an evaluation of social cognition, and the assessments of occupational functioning and subjective cognitive impairment were relatively rudimentary. Finally, in our study, cognitive impairment was defined by comparison to a healthy control group. Future research may benefit from more comprehensive assessments of social cognitive function and subjective cognitive impairment, such as the Perceived Deficits Questionnaire for Depression (PDQ-D) (96). Future studies could further explore whether threshold-based criteria offer distinct advantages in reflecting functioning and predicting outcomes in MDD patients compared to data-driven approaches. Beyond clinical considerations, the subtype concept should be further validated in mechanistic studies, incorporating biological markers such as glucose, lipids, inflammatory indices, and neuroimaging. Additionally, further investigation into the cognitive effects of benzodiazepines is needed, supported by an expanded sample size. Longer follow-up periods are also needed to evaluate the stability of cognitive subtypes. It is important to recognize that cognitive symptoms should be considered a clinically significant treatment target, as improving affect alone is not sufficient for achieving functional or lasting recovery.

## Data Availability

The datasets presented in this article are not readily available due to the inclusion of patients’ personal health information. Requests to access the datasets should be directed to CX, xuchenyang@stu.pku.edu.cn.
