# Supplementary material for: Parsing the heterogeneity of depression: a data-driven subgroup derived from cognitive function
Source: Front Psychiatry. 2025 Jan 30;16:1537331. doi: 10.3389/fpsyt.2025.1537331 (PMC11821656; doi:10.3389/fpsyt.2025.1537331)
Supplement: Supplementary file 1 [file DataSheet1.docx]

Supplementary Material

# Supplementary Methods

**Cognitive assessment tool: C-BCT**

(1) Trail Making Test, Part A (TMT-A): the respondent is required to connect consecutive numbers that are arranged in irregular locations on the electronic screen. If the respondent makes a mistake, the system will automatically recognize, remind him/her and then continue. The score represents the time taken to complete the task. This test mainly measures the cognitive areas involving the speed of information processing, visual scanning, and cognitive flexibility.

(2) Symbol Coding: in this test, the oracle bone scripts are used as symbols, which also symbolized Chinese cultural characteristics. The respondent is required to pair the numbers with corresponding oracle symbols as quickly as possible for a 90-s period. The number of correct answers will be counted as the total score. This test mainly measures attention, the speed of information processing, and the executive function of transformation.

(3) Continuous Performance Test (CPT): in this test, the Chinese zodiac was used as stimulus-responsive materials, as it is more suitable for the Chinese cultural environment. A total of three groups of different animal combinations are briefly and continuously played on the electronic device. The respondent is required to click each time that two stimuli in a row are identical. D-prime is used as the main evaluation index, which is a standard score that reflects the ability of subjects to distinguish between targets and distractions. This test mainly measures sustained and focused attention.

(4) Digit Span: this test adopts the form of voice broadcast with keystroke answers, which makes the operation simple and feasible. Ranging from two to nine digits, sequences of increasing length are played at a certain speed. For Digit Span Forward, the respondent is required to type the sequence of numbers in the same order as they are just played. For Digit Span Backward, the respondent is required to type the sequence of numbers in reverse order. This test mainly reflects the ability of auditory verbal working memory.

In a previous study, we reported the Chinese population norm for CBC-T. Stratified according to age, gender, and educational level, healthy adult subjects were recruited from fifteen institutions in seven administrative regions of China and 723 valid samples were obtained, of which 50 were retested. The Cronbach α of C-BCT is 0.75, and the test-retest reliability (ICC) ranged from 0.62 to 0.76. Normative data of C-BCT were generated by gender, age and education, and the effects of these demographic factors were analyzed. It revealed good internal consistency and test-retest reliability of C-BCT(1).

To correct for demographic factors affecting cognitive performance, the raw score was transformed into a T-score in each test (Mean: 50; SD: 10) adjusted by Chinese norms.

**Statistical Analysis**

**Choosing K-means Over Methods like Cut-off Scores:**

Although neurocognitive deficits are observed in patients with MDD, their severity is notably milder compared to those found in patients with schizophrenia or bipolar disorder(2). These findings suggest a gradient in the severity of neurocognitive impairments across schizophrenia, bipolar disorder, and MDD. A meta-analysis suggests performance deficits in executive functions seen in patients with major depressive disorder without psychotic features varying between 0.5 and 1.5 standard deviations below the performance of healthy controls(3).As the C-BCT was initially designed to assess cognition in patients with schizophrenia, using cut-off scores (e.g., -1 SD) may not be sensitive enough to adequately capture cognitive impairments in MDD patients.

**K-means Clustering**

We elected to use the scree plot elbow method and silhouette score as metrics for determining the optimal number of clusters for these are commonly used methods. The scree plot for a k-means clustering analysis shows the total within-cluster sum of squares (WSS) for each cluster for different values of k. The optimal number of clusters is often chosen at the "elbow" or bend in the scree plot, where the decrease in WSS slows down and the curve becomes less steep. Silhouette scores represent the mean silhouette coefficient over all instances of the dataset, and they range from-1 to 1. Scores closer to 1 indicate a model with more coherent clusters.

**Stepwise logistic regression analysis**

Before analysis, we converted categorical variables into dummy variables and performed collinearity tests. Pearson correlation coefficient was used for continuous variables, while Spearman correlation coefficient was used for ordinal variables. The variable "age of onset" was excluded due to collinearity with age (*r* = 0.868, *p* < 0.001). We employed the forward stepwise selection method based on likelihood ratio (LR) with the bias-corrected maximum likelihood estimation to identify the independent variables to be included in the model. Model fit was assessed using the Hosmer and Lemeshow test, and Cook's distance was used to detect outliers.

# Supplementary Figures and Tables

## Supplementary Figures





Supplementary Figure 1. Participant recruitment flow.


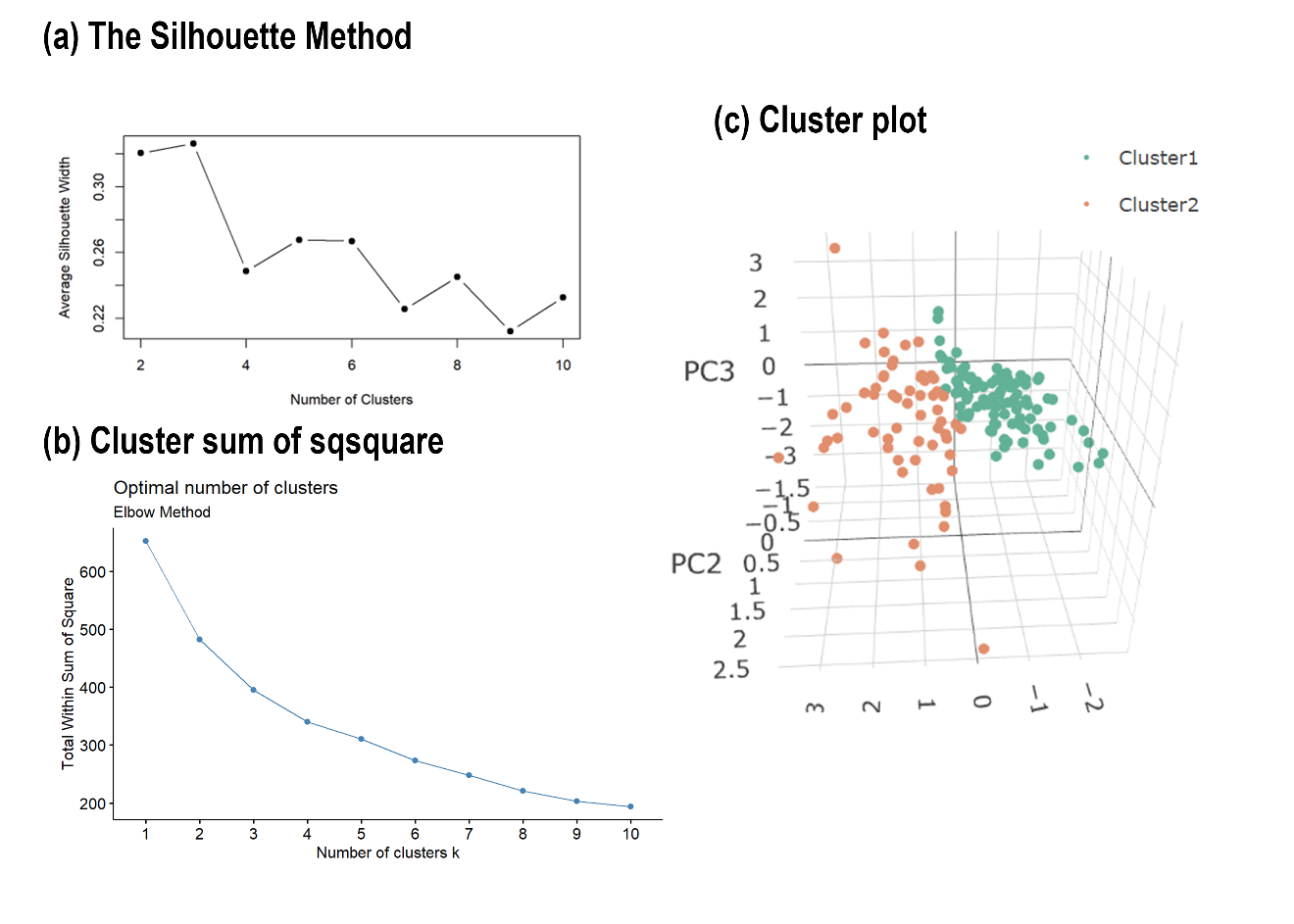


**Supplementary Figure 2. Deriving a Cognitive Subtype.** (a) The silhouette scores range from -1 to 1, with scores closer to 1 indicating a model with more coherent clusters; (b) The scree plot indicates an elbow at 2 clusters, after which the line begins to flatten;(c) Graphical clustering of patients with MDD using principal component analysis, showing that the 2 clusters are separable.

## Supplementary Tables

Supplementary Table 1. Demographics and clinical characteristics of MDD participants

| Measures | Total (n =163 ) | Follow-up subgroup(n=58) |
| --- | --- | --- |
| Sex, n (%) |  |  |
| Female | 104(64%) | 44(75%) |
| Male | 59(36%) | 15(25%) |
| Age, y | 29 (16) | 29(15) |
| Education level, y | 15(6) | 15(4) |
| HRSD-17 score | 19.66±4.99 | 19.51±5.0 |
| HAM-A score | 19.43±5.84 | 20.04±5.0 |
| FED, n (%) | 96(58.9%) | 34(58.6%) |

Data are n (%), mean ± SD, or median (IQR); MDD, major depressive disorder; FED, First Episode of Depression; HRSD-17, the 17-item Hamilton Rating Scale for Depression; HAM-A, Hamilton Rating Scale for Anxiety.

Supplementary Table 2. Comparison between two subgroups across clinical characteristics, treatment outcome and cognitive function at 8 Weeks

|  | Cognitive-impaired subgroup  n=24 | Cognitive-preserved subgroup  n=34 | Significance |
| --- | --- | --- | --- |
| Baseline | | | |
| HRDS-17 | 20.88±4.01 | 18.26±5.08 | F=4.389, p = 0.041 |
| HAM-A | 19.92±4.01 | 19.59±5.32 | F=0.065, p = 0.799 |
| Stop working | 6(25%) | 5(14.7%) | χ2 = 0.970, p = 0.325 |
| TMT-A | 43.14±5.15 | 48.56±2.53 | F=28.334,P＜o.001 |
| Digit Span | 38.96±10.83 | 50.91±7.71 | F=24.159,P＜o.001 |
| CPT | 40.83±9.84 | 51.41±3.83 | F=32.514,P＜o.001 |
| Symbol Coding | 41.67±9.00 | 51.59±9.03 | F=17.047,P＜o.001 |
| Final（8-weeks） | | | |
| HRSD-17 | 9.21±5.85 | 8.06±6.47 | F=0.481, p = 0.491 |
| Stop working | 1(4%) | 0 | χ2 = 1.442,p = 0.230 |
| TMT-A | 46.67±6.00 | 49.44±2.72 | F=5.649, P=0.021 |
| Digit Span | 40.38±10.45 | 53.15±6.58 | F=32.587, P＜o.001 |
| CPT | 47.33±9.85 | 52.03±2.96 | F=6.894, P=0.011 |
| Symbol Coding | 45.21±9.15 | 52.94±9.38 | F=9.758, P=0.003 |
| Remission  (HRDS-17＜7) | 12（50%） | 22（64.7%） | χ2 = 1.254, p = 0.263 |
| Type of medications | | | |
| Antidepressant dose(mg) | 36.75±20.26 | 39.19±18.64 | F = 0.225, p = 0.637 |
| Antipsychotic | 6(25%) | 6(17.6%) | χ2 = 0.464, p = 0.496 |
| Benzodiazepines | 4(16.6%) | 13(38.2%) | χ2 = 3.159, p = 0.076 |

Data are n (%), mean ± SD, or median (IQR); MDD, major depressive disorder; FED, First Episode of Depression; HRSD-17, the 17-item Hamilton Rating Scale for Depression; HAM-A, Hamilton Rating Scale for Anxiety; Antidepressant dose (mg): fluoxetine equivalents dose (mg).

Supplementary Table 3. Comparison between two subgroups across age and education level

|  | Cognitive-impaired subgroup  N=66 | Cognitive-preserved subgroup  N=97 |
| --- | --- | --- |
| Age, y | | |
| 18-29 | 26（39.40%） | 63（64.95%） |
| 30-39 | 21（31.82%） | 19（19.59%） |
| 40-49 | 9（13.64%） | 10（10.31%） |
| 50-59 | 10（15.15%） | 5（5.15%） |
| Education level, y | | |
| ＜10 | 27（40.91%） | 13（13.40%） |
| 10-12 | 11（16.67%） | 13（13.40%） |
| ＞12 | 28（42.42%） | 71（73.20%） |

Supplementary Table 4. Comparison of various commonly used cognitive assessment batteries.

|  | The Chinese Brief Cognitive Test (C-BCT) (1) | The MATRICS Consensus Cognitive Battery (MCCB)(4–6) | The Cambridge Neuropsychological Test Automated Battery (CANTAB) (7,8) | The Brief Assessment of Cognition in Affective Disorders (BAC-A)(9,10) |
| --- | --- | --- | --- | --- |
| Target Population | C-BCT is primarily designed for patients with schizophrenia, has also begun to be used in the study of affective disorders. | MCCB is primarily designed for patients with schizophrenia, now is among the most frequently used and most comprehensive neurocognitive assessment battery in clinical trials of psychiatric disorders. | CANTAB is a widely used neuropsychological assessment across diverse clinical conditions (e.g., mood disorders, traumatic brain injury, and ADRD) and study settings globally | BAC-A is an extension of the Brief Assessment of Cognition in Schizophrenia (BACS).And it is primarily used for patients with affective disorders. |
| Included Tasks | 1.TMT-A  2.Symbol Coding  3.Continuous Performance Test  4.Digit Span | 1. Speed of Processing (Symbol Coding)  2. Attention/Vigilance (Continuous Performance Test, CPT)  3. Working Memory (Digit Span)  4. Verbal Learning (Hopkins Verbal Learning Test, HVLT)  5. Visual Learning (Brief Visuospatial Memory Test, BVMT)  6. Reasoning & Problem-Solving (NAB)  7. Social Cognition (Mayer-Salovey-Caruso Emotional Intelligence Test, MSCEIT)  8. Reasoning (Matrix Reasoning Test)  9. Executive Function (Trail Making Test, TMT) | 1. Delayed Matching to Sample (DMS)  2. Spatial Working Memory (SWM)  3. Stop Signal Task (SST)  4. Iowa Gambling Task (IGT)  5. Paired Associates Learning (PAL)  6. Rapid Visual Information Processing (RVIP)  7. Odd One Out (OO)  8. Continuous Performance Task (CPT) | 1.Token Motor Task  2.Symbol Coding  3.List Learning  4.Digit Sequencing Task  5.Category Instances (Animals) and Controlled Oral Word Association Test (F and S-words)  6.Tower of London  7.The Emotion Inhibition Test (a modified version of the Emotional Stroop task )  8.The affective auditory verbal learning test (Affective interference test) |
| Cognitive Domains Assessed | Attention, working memory, speed of processing, cognitive flexibility, the executive function of transformation | Speed of processing, attention/vigilance, working memory, verbal learning, visual learning, reasoning and problem solving, social cognition | Attention, working memory, processing speed, verbal learning, visual learning, reasoning, social cognition, executive function | Verbal memory, working memory, motor speed, verbal fluency, attention and processing speed, executive function, and emotional cognition |
| Test Type | APP-based online tasks to support devices such as cell phones, tablets and computers. | Paper-and-pencil test | Computerized test, requires a dedicated computer or touch screen device | Paper-and-pencil test |
| Testing Time | 15min | About 60-90min | Each task takes about 10-20 minutes, and the entire battery takes approximately 40-60 minutes | About 40min |
| Application in Chinese population | Developed for the Chinese population, completed the reliability test and established normative data for C-BCT by gender, age, literacy and other statistical factors | YES | YES | NO |

**Reference**

1. Ye S, Xie M, Yu X, Wu R, Liu D, Hu S, Xu Y, Liu H, Wang X, Zhu G, et al. The Chinese Brief Cognitive Test: Normative Data Stratified by Gender, Age and Education. *Front Psychiatry* (2022) 13:933642. doi: 10.3389/fpsyt.2022.933642

2. Terachi S, Yamada T, Pu S, Yokoyama K, Matsumura H, Kaneko K. Comparison of neurocognitive function in major depressive disorder, bipolar disorder, and schizophrenia in later life: A cross-sectional study of euthymic or remitted, non-demented patients using the Japanese version of the Brief Assessment of Cognition in Schizophrenia (BACS-J). *Psychiatry Res* (2017) 254:205–210. doi: 10.1016/j.psychres.2017.04.058

3. Wagner S, Doering B, Helmreich I, Lieb K, Tadić A. A meta-analysis of executive dysfunctions in unipolar major depressive disorder without psychotic symptoms and their changes during antidepressant treatment. *Acta Psychiatrica Scandinavica* (2012) 125:281–292. doi: 10.1111/j.1600-0447.2011.01762.x

4. Kh N, Mf G, Rs K, Le B, Dm B, Jd C, S E, Ws F, Fj F, Jm G, et al. The MATRICS Consensus Cognitive Battery, part 1: test selection, reliability, and validity. *The American journal of psychiatry* (2008) 165: doi: 10.1176/appi.ajp.2007.07010042

5. Burdick KE, Goldberg TE, Cornblatt BA, Keefe RS, Gopin CB, Derosse P, Braga RJ, Malhotra AK. The MATRICS consensus cognitive battery in patients with bipolar I disorder. *Neuropsychopharmacology* (2011) 36:1587–1592. doi: 10.1038/npp.2011.36

6. Russo M, Mahon K, Burdick KE. Measuring cognitive function in MDD: emerging assessment tools. *Depress Anxiety* (2015) 32:262–269. doi: 10.1002/da.22297

7. Rock PL, Roiser JP, Riedel WJ, Blackwell AD. Cognitive impairment in depression: a systematic review and meta-analysis. *Psychol Med* (2014) 44:2029–2040. doi: 10.1017/S0033291713002535

8. Rhee TG, Shim SR, Manning KJ, Tennen HA, Kaster TS, d’Andrea G, Forester BP, Nierenberg AA, McIntyre RS, Steffens DC. Neuropsychological Assessments of Cognitive Impairment in Major Depressive Disorder: A Systematic Review and Meta-Analysis with Meta-Regression. *Psychotherapy and Psychosomatics* (2024) 93:8. doi: 10.1159/000535665

9. Jia X, Wang T, Han H, Liu J, Wang L, Tian B, Wang C. Correlation of cognitive function and clinical characteristics in adolescent depressive disorder patients with self-injury behavior. *Chinese Journal of Behavioral Medicine and Brain Science* (2023)707–713.

10. J Z, J X, R L, H Q, J Y, T G, J Z, X Z, L Z, X C, et al. A prospective cohort study of depression (PROUD) in China: rationale and design. *Current medicine (Cham, Switzerland)* (2023) 2: doi: 10.1007/s44194-022-00018-7
